# Prevalence, Associated Risk Factors, Farmers' Knowledge, and Farm Management Practices of Calf Coccidiosis in and Around Gondar City, Northwest Ethiopia

**DOI:** 10.1002/vms3.71326

**Published:** 2026-11-05

**Authors:** Nibretu Tsegaye Guto, Yohannes Balework Zeleke, Gebremikael Asnakew Wubie, Bezawit Kassaw Hailu, Ambaye Kenubih

**Affiliations:** ^1^ Department of Biomedical Sciences College of Veterinary Medicine and Animal Sciences University of Gondar Gondar Ethiopia; ^2^ Department of Pathobiology College of Veterinary Medicine and Animal Sciences University of Gondar Gondar Ethiopia

**Keywords:** calves, coccidiosis, Eimeria, Gondar, prevalence, risk factors

## Abstract

**Background:**

Coccidiosis is an important enteric disease of calves caused by protozoan parasites of the genus *Eimeria*. Despite its impact on calf health, information on its occurrence, associated factors, and farm‐level management practices is limited in and around Gondar City, Northwest Ethiopia. This study was conducted to estimate the prevalence of calf coccidiosis, identify factors associated with *Eimeria* infection, and assess farmers' knowledge and farm management practices.

**Methods:**

A cross‐sectional study was conducted from December 2024 to June 2025. A two‐stage sampling procedure was employed to collect 256 faecal samples from calves up to 1 year of age across 71 randomly selected farms. Samples were examined for *Eimeria* oocysts using a flotation technique with saturated salt solution. Data were summarized using descriptive statistics, and factors associated with *Eimeria* infection were assessed using logistic regression analysis in SPSS version 30.

**Results:**

The overall prevalence of coccidiosis was 29.7% (76/256) at calf‐level and 59.2% (42/71) at farm‐level. Multivariable logistic regression analysis showed that age ≤6 months (AOR = 5.73), poor body condition (AOR = 14.46), herd size >50 animals (AOR = 9.79), intensive farming (AOR = 7.34), river water (AOR = 10.24), and soil flooring (AOR = 6.81) were significantly associated with higher odds of Eimeria infection (*p* < 0.05). In contrast, non‐weaned calves had significantly lower odds of infection compared to weaned calves (AOR = 0.19; *p* = 0.011). Assessment of farm practices showed that 84.5% of the farm owners had no knowledge of calf coccidiosis, improper waste disposal was practiced on 53.5% of the farms, and inadequate implementation of control measures was observed on 54.9% of the farms.

**Conclusions:**

The findings indicate that calf coccidiosis is an important health problem in the study area and emphasize the need for improved farm management, enhanced farmer knowledge, and effective control strategies.

## Graphical abstract

This study found a high prevalence of calf coccidiosis in and around Gondar City, Ethiopia, with significant associations between infection and management‐related risk factors such as age, body condition, herd size, water source, and housing conditions, highlighting the need for improved control measures and targeted prevention strategies.

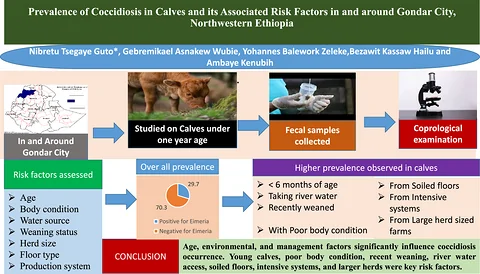

## 1 Introduction

Bovine coccidiosis is a globally important enteric disease of cattle, caused by protozoan parasites belonging to the genus *Eimeria* (Daugschies and Najdrowski 2005). The disease imposes heavy economic losses on both dairy and beef operations worldwide (Sarfaraz et al. 2025). In cattle, more than 20 *Eimeria* species have been described, of which *Eimeria bovis* and *Eimeria zuernii* are the most pathogenic and account for the majority of clinical outbreaks, particularly in young animals (Olivares‐Muñoz et al. 2022). Calves up to 1 year of age are especially vulnerable because their immune systems are not yet fully mature, increasing the risk of both clinical and subclinical disease (Lopez‐Osorio et al. 2020). Clinical coccidiosis is characterized by acute damage to the intestinal mucosa, resulting in diarrhoea (often haemorrhagic), dehydration, anorexia, weight loss, and, in severe cases, mortality (Tomczuk et al. 2015). Subclinical infections, though less apparent, are equally detrimental to productivity, leading to reduced feed conversion efficiency, poor weight gain, and compromised long‐term performance, ultimately affecting age at first calving and lifetime milk yield (Ekawasti et al. 2022).

Transmission occurs when calves ingest sporulated oocysts from contaminated feed, water, or bedding (Olivares‐Muñoz et al. 2022). The epidemiology of bovine coccidiosis is complex and shaped by multiple interacting factors (Tamrat et al. 2020). Among host‐related variables, age stands out as the most consistent predictor of infection risk; calves aged 1–6 months are most susceptible, with resistance gradually developing as they grow older. Management practices are equally critical. Poor pen hygiene, infrequent manure removal, high stocking densities, and housing calves together with adults all promote oocyst build‐up in the environment and increase infection pressure (Das et al. 2015; Mathewos and Endale 2024). Stressful events such as weaning, dehorning, transport, or abrupt diet changes can also trigger clinical disease by temporarily suppressing immunity (Razavi et al. 2024). Environmental conditions also influence coccidiosis occurrence, as warm and humid climates favour oocyst sporulation and survival, contributing to higher prevalence during rainy seasons (Adinehbeigi et al. 2018). The combined effect of these risk factors explains why the disease prevalence varies so widely across farms, regions, and production systems (Shamsi et al. 2026; Disfani et al. 2025).

In Ethiopia, livestock production is central to the national economy and supports millions of smallholder farmers. However, the sector faces major constraints from infectious and parasitic diseases, which remain leading causes of substantial losses in animal health and productivity (Tsouloufi et al. 2023). Coccidiosis is among these constraints and continues to affect productivity, especially in intensive and semi‐intensive calf rearing systems. Several studies from different parts of Ethiopia have documented calf coccidiosis, with prevalence figures ranging from 18% to over 70% depending on location, season, and management (Tamrat et al. 2020; Mathewos and Endale 2024; Alemayehu et al. 2013; Gebeyehu et al. 2018; Ambaw et al. 2025). Although these studies have investigated the prevalence and associated risk factors in various parts of the country, data from Gondar and its surroundings remain limited. To date, only one study exists, and it was conducted more than 7 years ago (Alemie and Duguma 2020). Since then, farming practices have changed, livestock production systems have intensified in some areas, and environmental conditions have shifted, all of which could alter disease patterns. Furthermore, none of the previous studies in this area have examined farm management practices or farmers' awareness related to calf coccidiosis, which are important components for understanding disease prevention and control at the farm level. This is a notable gap because effective disease control depends not only on understanding biological risk factors but also on addressing how farmers manage their animals and perceive the disease. Therefore, the present cross‐sectional study was designed to estimate the current prevalence of *Eimeria* infection in calves up to 1 year of age; to identify the host, management, and environmental factors associated with infection; and to assess farm owners' awareness and management practices regarding calf coccidiosis in and around Gondar City, Northwest Ethiopia.

## 2 Materials and Methods

### 2.1 Study Area

The study was conducted in and around Gondar City from December 2024 to June 2025. Gondar City is located in the Amhara Regional State of northwestern Ethiopia (Figure 1). The study area lies between 12.30° and 13.80°N latitude and 35.30° and 35.70°E longitude, with an average altitude of approximately 2220 m above sea level. The city is approximately 727 km from Addis Ababa, the capital city of Ethiopia. The mean annual rainfall is 1172 mm, and the average annual temperature is 19.7°C (Taddese et al. 2021).

**FIGURE 1:**
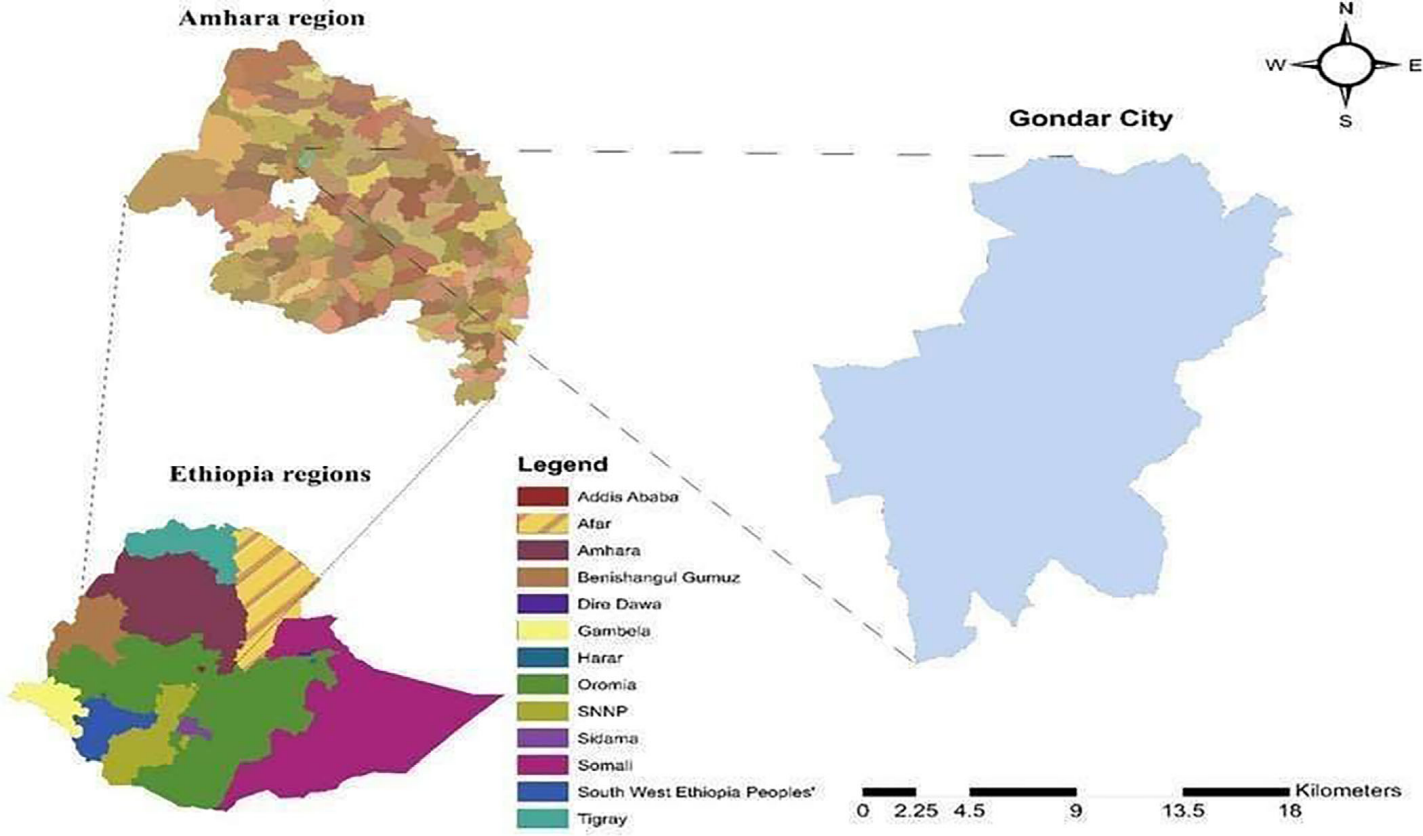
Map of the study area showing the location of Ethiopia within Africa (inset), Ethiopia with the Amhara Region and Gondar City, and an enlarged view of Gondar City and surrounding districts where faecal samples were collected from dairy and beef farms. SNNPR, Southern Nations, Nationalities, and Peoples’ Region.

The predominant production system in the region is mixed crop‐livestock farming, and cattle are the most important livestock species reared in this region (Worku et al. 2018). The study area encompasses three main cattle production systems: extensive, semi‑intensive, and intensive. Under the extensive system, cattle graze communally with little or no housing, and calves are kept alongside adults with minimal intervention. Semi‑intensive farms provide partial housing and daytime grazing with some supplementary feeding. Intensive operations, by contrast, keep cattle housed year‑round under zero‑grazing systems, relying on concentrate feeds and improved water supply (Food and Agriculture Organization of the United Nations 2017). The study area experiences a shortage of tap water, and many farms rely on river water for their cattle, including intensive farms where water is fetched to housed calves. In the highlands of North Gondar, mixed crop‑livestock and transhumance systems are also common; during the rainy season, many farmers move cattle to lowland areas where feed and grazing land are more readily available (Tegegne et al. 2009).

According to the Gondar City Administration Livestock Development Office (unpublished data), the city administration comprises 11 rural and 25 urban kebeles and has more than 600 cattle owners/handlers. In 2024, the estimated cattle population was approximately 91,000, of which about 61,000 were local breeds and 29,100 were crossbreeds.

### 2.2 Study Population

The study population comprised both male and female calves up to 1 year of age. Calves were divided into two age categories: up to 6 months and >6–12 months. The ages were determined using records from animal history logs maintained on intensive dairy farms, as well as through information obtained from farm owners. Based on breed, calves were classified as local or crossbred, determined through owner information and physical inspection. Body condition score was assessed using the criteria described by Nicholson and Butterworth (1986) (Supporting Information 1), with calves categorized as poor, medium, or good. The scoring system was originally developed for zebu cattle and was considered appropriate for the local breeds included in this study. A similar 1–9 scale was later developed for dairy cattle (Edmonson et al. 1989). Although these systems were primarily developed for adult cattle, modified body condition scoring approaches based on palpation have been applied to calves (Raschke 2007). Therefore, in the present study, body condition was assessed through palpation of anatomical landmarks rather than visual appraisal alone, which is considered more appropriate for young animals where fat deposition patterns differ from mature cattle.

### 2.3 Sample Size Determination

The minimum sample size was calculated using the single population proportion formula described by Thrusfield and Christley (2018): *n* =$\;\frac{\;p\;\times \left(1-p\right){\left(Z\alpha /2\right)}^{2}}{d^{2}}$, with the following assumptions: expected prevalence (*p*) of 21.1% from a previous study in Gondar City (Alemie and Duguma 2020), 95% confidence level (*Z* = 1.96), and 5% margin of error (*d*). Accordingly, the minimum sample size (*n*) was found to be 256 calves.

### 2.4 Study Design and Sampling Technique

A cross‐sectional study was conducted from December 2024 to June 2025 using a two‐stage sampling procedure. In the first stage, cattle‐owning households (farms) were randomly selected from the sampling frame (600) obtained from the Gondar City Administration Livestock Development Office. Selected farms were visited and screened for eligibility based on the presence of at least one calf up to 1 year of age and not treated with anticoccidials within the previous 2 weeks. Farms without eligible calves were excluded, and additional farms were randomly selected from the remaining sampling frame until sufficient farms were obtained. For the purpose of this study, a farm was defined as a household‐level production unit managed by an individual owner.

In the second stage, all eligible calves within the selected farms were identified through direct animal inventory. The number of calves sampled from each farm was determined using proportional allocation based on the number of eligible calves available on each farm. Within farms with more than one eligible calf, calves were selected using a simple random sampling method. Farms with only one eligible calf contributed that calf to the study. Farm selection and calf sampling continued until the required sample size of 256 calves was achieved, resulting in the inclusion of 71 farms. Farms were categorized as extensive (*n* = 20), semi‐intensive (*n* = 15), or intensive (*n* = 36) based on the production system definitions described in Section 2.1.

### 2.5 Sample Collection

A fresh faecal sample of approximately 20 g was collected directly from the rectum of each calf using a sterile universal container. Each sample was placed in a clean, labelled glass bottle and transported in an ice box with ice packs to the Gondar University Veterinary Parasitology Laboratory on the same day. Samples were stored at 4°C in a refrigerator and processed within 48 h of arrival (Modrý et al. 2017). During sample collection, relevant information, including the owner's name, date of sampling, age, sex, breed, body condition score, and tag number (if available), was recorded for each calf using a structured data recording sheet (Supporting Information 2). Weaning status was assessed through farmer interviews and calf management history. Calves were categorized as weaned if they had been separated from the dam and were no longer receiving milk, while calves still receiving milk were classified as non‐weaned.

### 2.6 Faecal Examination

A simple flotation technique was applied to concentrate coccidian oocysts. Briefly, approximately 3 g of faecal material was placed in a beaker, followed by the addition of 42 mL of saturated salt (sodium chloride) flotation solution. The mixture was thoroughly stirred to obtain a homogeneous faecal suspension, which was then poured through a tea strainer into a second beaker. From this beaker, the suspension was transferred into a test tube, which was gently filled to form a convex meniscus at the top. A coverslip was carefully placed over the opening of the test tube to trap oocysts on the surface, and the setup was allowed to stand for 20 min (the detailed procedure is provided in Supporting Information 3). After this period, the coverslip was removed and placed on a microscope slide for examination under a microscope (Zajac et al. 2021). A photomicrograph of the detected *Eimeria* oocyst is presented in Supporting Information 4.

### 2.7 Assessment of Farm Management Practices

A semi‐structured questionnaire (Supporting Information 5) was administered to 71 farm owners available during the sampling period to assess farm management practices and farmers’ knowledge of calf coccidiosis. The questionnaire covered hygiene practices, waste disposal, calf housing, colostrum feeding, history and treatment of diarrhoea, calf–adult cattle contact, preventive measures, and owner‐related characteristics, including education level, farming experience, training status, and animal‐related educational background. Additional information was obtained through direct observation of farm conditions. Facility‐based indicators, including ventilation, footbaths, isolation facilities, housing separation, overcrowding, and floor condition, were assessed for descriptive purposes only on semi‐intensive and intensive farms where housing facilities were present (*n* = 51). For analysis of factors associated with *Eimeria* infection, calf–adult cattle contact, overcrowding, housing separation, and floor type were operationally classified for calves across all three production systems. In extensive farms, calves were classified as having contact with adult cattle because they shared grazing areas with adult animals, as not overcrowded because they were not confined in housing structures, as unseparated because separate calf housing was absent, and as having soil floors because they were maintained on natural ground. These operational classifications enabled the inclusion of these variables in the association analysis across all production systems, although the corresponding facility‐based conditions were not directly observed in extensive farms.

### 2.8 Data Management and Statistical Analysis

Data collected from each examined calf, including laboratory results, animal characteristics, and management‐related factors, were recorded in a Microsoft Excel database. The dataset was cleaned, coded, and summarized using descriptive statistics, including frequencies, proportions, and percentages. Statistical analysis was performed using the Statistical Package for the Social Sciences (SPSS) version 30. Calf‐level prevalence was calculated as the proportion of *Eimeria*‐positive calves among all examined calves, while farm‐level prevalence was calculated as the proportion of farms with at least one positive calf. Associations between explanatory variables and *Eimeria* infection status were assessed using univariable and multivariable logistic regression. Variables with a *p* value ≤0.25 and without evidence of collinearity were included in the multivariable logistic regression model. This selection criterion was used to retain potentially important variables and minimize the effect of confounding. Multicollinearity among explanatory variables was evaluated using variance inflation factors (VIFs) and tolerance values; variables with VIF values <5 were considered free from problematic multicollinearity. The goodness‐of‐fit of the final multivariable logistic regression model was assessed using the Hosmer–Lemeshow test, with a *p* value >0.05 indicating adequate model fit (Audige 2005). Statistical significance was declared at *p* < 0.05.

## 3 Results

### 3.1 Demographic Characteristics of Farm Owners

A total of 71 farm owners were surveyed in the study area. Most farm owners were male (71.8%), and the majority had primary education or above (60.6%). Farms were typically managed by the owners themselves (97.2%). Although over half of the respondents (63.4%) had received training related to calf management, very few had an animal‐related academic background. In terms of experience, almost half (47.9%) had less than 10 years of farm experience, while 52.1% had 10 years or more (Table 1).

**TABLE 1:** Demographic characteristics of farm owners in and around Gondar City (*n* = 71).

Owner characteristic	Category	Frequency	Percentage
Sex of owners	Male	51	71.8
	Female	20	28.2
Level of education	Illiterates	28	39.4
	Primary and above	43	60.6
Person who manages the farm	Owner	69	97.2
	Employer	2	2.8
Owner received training	Yes	45	63.4
	No	26	36.6
Years of farm experience	<10	34	47.9
	>10	37	52.1

### 3.2 Assessment of Farm Management Practices and Farmers Knowledge

Assessment of the 71 farms revealed notable gaps in calf management practices and disease awareness. Feeding and watering hygiene and colostrum feeding were practiced on more than half of the farms, whereas proper waste disposal and general control measures were less commonly practiced. Awareness of coccidiosis and calf vaccination among farm owners was low, despite a high reported history of diarrhoea. Details are presented in Table 2.

**TABLE 2:** Assessment of calf management practices, disease awareness, and preventive measures among farms in and around Gondar City (*n* = 71).

Parameter	Yes, n (%)	No, n (%)
Feeding and watering hygiene practised	39 (54.9)	32 (45.1)
Proper waste disposal practiced	33 (46.5)	38 (53.5)
Knowledge of coccidiosis	11 (15.5)	60 (84.5)
History of diarrhoea on the farm	70 (98.6)	1 (1.4)
Diarrhoea treatment provided	54 (76.1)	17 (23.9)
General control measures implemented	32 (45.1)	39 (54.9)
Owner awareness of calf vaccination	15 (21.1)	56 (78.9)
Colostrum feeding practiced	53 (74.6)	18 (25.4)

### 3.3 Researcher's on‐Site Observational Evaluation

The researcher's observations on semi‑intensive and intensive farms (n = 51) are summarized in Table 3. Isolation facilities were lacking on most farms (84.5%), and footbaths were present on very few farms (9.9%). Overcrowding was observed on nearly 40% of farms, and rough or wet floors were common (63.4%).

**TABLE 3:** Findings based on the researcher's observation on semi‑intensive and intensive farms in and around Gondar City (*n* = 51).

Farm assessment indicator	Yes, n (%)	No, n (%)
Isolation facility for sick animals	8 (15.7)	43 (84.3)
Housing partitioned by age group	35 (68.6)	16 (31.4)
Calf–adult cattle contact	18 (35.3)	33 (64.7)
Adequate ventilation	37 (72.5)	14 (27.5)
Footbath at the farm entrance	5 (9.8)	46 (90.2)
Overcrowding of cattle	20 (39.2)	31 (60.8)
Pen floor condition (rough or wet)	32 (62.7)	19 (37.3)

### 3.4 Farm Level Prevalence

Out of the 71 total farms surveyed, 42 farms (59.2%) had at least one calf that tested positive for coccidiosis, while 29 farms (40.8%) had no positive cases. This indicates that calf coccidiosis was present in more than half of the farms included in the study.

### 3.5 Prevalence of *Eimeria* Infection

Out of 256 faecal samples tested, 76 were positive for *Eimeria* oocysts, resulting in an overall calf‐level prevalence of 29.7% (95% CI: 24.2%–35.8%). When considering host‐related factors (Table 4), the prevalence of *Eimeria* infection varied across the different categories. Higher prevalence was observed among calves aged ≤6 months (36.9%), those with poor body condition (65.3%), weaned calves (43.3%), and calves from herds with more than 50 animals (55.2%). The prevalence was relatively similar between male (32.1%) and female calves (27.8%) and between local (36.8%) and crossbred calves (26.0%).

**TABLE 4:** Prevalence of calf coccidiosis according to host‐related factors in and around Gondar City (*n* = 256).

Variables	Category	Total examined, n	Positives, n (%)
Sex	Male	112	36 (32.1)
	Female	144	40 (27.8)
Breed	Local	87	32 (36.8)
	Cross	169	44 (26.0)
Age	≤6 months	141	52 (36.9)
	>6–12 months	115	24 (20.9)
Body condition	Good	86	6 (7.0)
	Medium	98	23 (23.5)
	Poor	72	47 (65.3)
Weaning status	Weaned	90	39 (43.3)
	Non‐weaned	166	37 (22.3)
Herd size	<10 animals	119	28 (23.5)
	10–50 animals	108	32 (29.6)
	>50 animals	29	16 (55.2)
Total		256	76 (29.7)

Management and environmental factors were assessed in relation to calf coccidiosis prevalence (Table 5). Prevalence varied across farming systems, with higher infection observed in intensively managed calves. Higher prevalence was also observed in unseparated housing, overcrowded conditions, and among calves with soil flooring. In contrast, prevalence was similar between calves from farms where proper colostrum feeding was practiced (29.7%) and those from farms where it was not practiced (29.3%). Water source also showed variation in prevalence, with the highest infection observed among calves provided with river water. The distribution of water sources across production systems showed that river water was used in all systems: all 20 extensive farms, seven of 15 semi‑intensive farms, and 16 of 36 intensive farms, reflecting tap water shortages in the area. Across all calves, 151 used river water, 30 used underground water, and 75 used tap water. The VIF indicated no substantial multicollinearity between farming system and water source (VIF = 1.59).

**TABLE 5:** Prevalence of calf coccidiosis according to farm management and environmental factors in and around Gondar City (*n* = 256).

Variable	Category	Calves examined, n	Positives, n (%)
Farming system	Extensive	63	9 (14.3)
	Semi‐intensive	53	9 (17.0)
	Intensive	140	58 (41.4)
Proper colostrum feeding	Yes	192	56 (29.2)
	No	64	20 (31.3)
House condition	Separated	202	54 (26.7)
	Unseparated	54	22 (40.7)
Water source	Tap water	75	11 (14.7)
	Underground	30	9 (30.0)
	River	151	56 (37.1)
Calf–adult cattle contact	Yes	202	60 (29.7)
	No	54	16 (29.3)
Floor type	Concrete	146	35 (24.0)
	Soil	110	41 (37.3)
Overcrowding	Yes	107	40 (37.4)
	No	149	36 (24.2)
Total		256	76 (29.7)

### 3.6 Factors Associated With *Eimeria* Infection

In the univariable logistic regression analysis, more than 20 potential animal‐related and farm management and environmental factors were assessed for their association with Eimeria infection among calves. Animal‐related factors included age, breed, sex, body condition, and weaning status, whereas farm management and environmental factors included farming system, total herd size, housing separation, overcrowding, floor type, and water source. These factors were considered epidemiologically relevant because farm management, housing conditions, and environmental conditions may influence faecal contamination and exposure to *Eimeria* oocysts. Variables with *p* < 0.25 in the univariable analysis were considered for inclusion in the multivariable logistic regression model.

Multicollinearity among the predictor variables was assessed using the VIF. All variables included in the final model had VIF values below 5, ranging from 1.18 to 1.98, indicating no evidence of problematic multicollinearity. The Hosmer–Lemeshow goodness‐of‐fit test showed a *χ* ^2^ value of 14.486 with 8 degrees of freedom and a *p* value of 0.070, indicating an adequate fit of the final model.

The final multivariable logistic regression model identified several factors significantly associated (*p* < 0.05) with *Eimeria* infection (Table 6). Calves aged ≤6 months had higher odds of infection than those aged >6–12 months (AOR = 5.73). Compared with calves in good body condition, those in medium and poor body condition had significantly higher odds of infection (AOR = 3.57 and 14.46, respectively). Non‐weaned calves had lower odds of infection than weaned calves (AOR = 0.19). Among the farm management and environmental factors, intensive farming systems (AOR = 7.34), herd sizes >50 animals (AOR = 9.79), soil flooring (AOR = 6.81), and river water sources (AOR = 10.24) were significantly associated with increased odds of *Eimeria* infection. In contrast, semi‐intensive farming, medium‐sized herds, underground water sources, housing separation, and overcrowding were not significantly associated (*p* > 0.05) with infection after adjustment for other variables in the model. Breed and sex were also not significantly associated with infection.

**TABLE 6:** Univariable and multivariable logistic regression analysis of factors associated with *Eimeria* infection among calves in and around Gondar City (*n* = 256).

		Test result		Univariate		Multivariate	
Variable	Category	Positive, n (%)	Negative, n (%)	COR (95% CI)	*p* value	AOR (95% CI)	*p* value
Age	>6–12 months	24 (20.9)	91 (79.1)	Ref	—	Ref	—
	≤6 months	52 (36.9)	89 (63.1)	2.22 (1.26–3.90)	0.006	5.73 (1.66–19.78)	0.006
Breed	Local	32 (36.8)	55 (63.2)	Ref	—	Ref	—
	Cross	44 (26.0)	125 (74.0)	0.61 (0.35–1.05)	0.076	0.36 (0.12–1.12)	0.078
Sex	Male	36 (32.1)	76(67.9)	Ref		—	—
	Female	40 (27.8)	104 (72.2)	0.81 (0.47–1.39)	0.449	—	—
Body condition	Good	6 (7.0)	80 (93.0)	Ref	—	Ref	—
	Medium	23 (23.5)	75 (76.5)	4.09 (1.58–10.59)	0.004	3.57 (1.06–12.07)	0.040
	Poor	47 (65.3)	25 (34.7)	25.07 (9.59–65.54)	<0.001	14.46 (4.11–50.81)	<0.001
Weaning status	Weaned	39 (43.3)	51 (56.7)	Ref	—	Ref	—
	Non‐weaned	37 (22.3)	129 (77.7)	0.38 (0.21–0.65)	<0.001	0.19 (0.06–0.69)	0.011
Farming system	Extensive	9 (14.3)	54 (85.7)	Ref	—	Ref	—
	Semi‐intensive	9 (17.0)	44 (83.0)	1.23 (0.45–3.36)	0.690	0.30 (0.06–1.45)	0.134
	Intensive	58 (41.4)	82 (58.6)	4.24 (1.94–9.27)	<0.001	7.34 (1.62– 33.21)	0.010
Total herd size	<10 animals	28 (23.5)	91 (76.5)	Ref	—	Ref	—
	10–50	32 (29.6)	76 (70.4)	1.37 (0.76–2.47)	0.299	1.87 (0.48–7.25)	0.364
	>50 animals	16 (55.2)	13 (44.8)	4.00 (1.72–9.32)	0.001	9.79 (1.42–67.46)	0.021
Housing separation	Separated	54 (26.7)	148 (73.3)	Ref	—	Ref	—
	Unseparated	22 (40.7)	32 (59.3)	1.88 (1.01–3.52)	0.047	1.59 (0.41–6.13)	0.498
Overcrowded	Yes	40 (37.4)	67 (62.6)	Ref	—	Ref	—
	No	36 (24.2)	113 (75.8)	0.53 (0.31–0.92)	0.023	1.34 (0.44– 4.02)	0.608
Floor type	Concrete	35 (24.0)	111 (76.0)	Ref	—	Ref	—
	Soil	41 (37.3)	69 (62.7)	1.88 (1.09–3.24)	0.022	6.81 (1.62–28.73)	0.009
Water source	Tap water	11 (14.7)	64 (85.3)	Ref	—	Ref	—
	Underground	9 (30.0)	21 (70.0)	2.49 (0.91–6.84)	0.076	3.49 (0.71–17.15)	0.123
	River	56 (37.1)	95 (62.9)	3.43 (1.67–7.05)	<0.001	10.24 (2.43–43.18)	0.002
Total		76 (29.7)	180 (70.3)				

Abbreviation: AOR, adjusted odds ratio; CI, confidence interval; COR, crude odds ratio; Ref, reference.

## 4 Discussion

### 4.1 Prevalence of Calf Coccidiosis

The overall prevalence of calf coccidiosis was 29.7% in the study area. This finding is consistent with several previous studies reporting nearly similar prevalence, including 31.0% in Jimma (Nuriye et al. 2020), 31.9% in Kombolcha (Alemayehu et al. 2013), 26.0% in and around Holeta (Regasa et al. 2018), and 30.7% in Sekota (Worku et al. 2019). Similarly, studies in Thailand reported a prevalence of 22.3% (Keomoungkhoun and Taweenan 2015). The prevalence observed in the present study was lower than those reported in previous studies conducted in Asela town (62.5%) (Asfaw et al. 2016), Arsi Zone (53.6%) (Mekonnin and Furgasa 2018), Mekelle (72.7%) (Gebeyehu et al. 2018), and Sebeta (57.5%) (Ashagrie et al. 2022). Similarly, higher prevalence was documented in other countries, including Poland (93.0%) (Klockiewicz et al. 2007), Colombia (75.5%) (Lopez‐Osorio et al. 2020), and Mexico (39.7%) (Olivares‐Muñoz et al. 2022). In contrast, the current finding reported a higher prevalence than reports from Bahir Dar (20.1%) (Adinehbeigi et al. 2018), Kacha Bira District (17.8%) (Tamrat et al. 2020), and Iraq (9.5%) (Hussin 2016). The differences in prevalence reported across studies may be attributable to variations in diagnostic methods, the age distribution of sampled calves, production systems, farm management practices, and sampling periods. Diagnostic sensitivity varies considerably among coprological techniques. The choice of flotation solution and its specific gravity can affect oocyst recovery, although within an optimal range, the specific gravity remains sufficient to float *Eimeria* oocysts effectively (Cruvinel et al. 2021). The timing of sample collection relative to seasonal peaks in oocyst shedding also influences prevalence estimates, as oocyst excretion varies by season and by age, with younger animals typically shedding higher numbers of oocysts (Lucas et al. 2014). In addition, differences in housing conditions, stocking density, and hygiene practices may affect the level of environmental contamination and calves’ exposure to infective oocysts (Radostits et al. 2010).

The farm‐level prevalence of calf coccidiosis in and around Gondar City was 59.2%. This finding was lower than the herd‐level prevalence reported from Sebeta Town (78.7%) and Dire Dawa (99.0%) in Ethiopia (Ashagrie et al. 2022; Dawid et al. 2012). Similarly, higher farm‐level prevalences have been reported outside Ethiopia, including 93.0% in Poland and 83.1% in Germany (Klockiewicz et al. 2007; Bangoura et al. 2012). These variations may be attributed to differences in environmental conditions, farmer awareness, management practices, production systems, and other factors influencing the transmission and distribution of coccidiosis (Adinehbeigi et al. 2018). The farm‐level prevalence of 59.2% was higher than the calf‐level prevalence of 29.7%, indicating that Eimeria infection was detected on a substantial proportion of surveyed farms, although not all calves were infected. These results highlight the importance of considering both animal‐level and farm‐level occurrence when planning surveillance and preventive interventions.

### 4.2 Associated Factors of Calf Coccidiosis

Among the factors analyzed, calf age showed a significant association with coccidial infection (*p* < 0.05). A higher prevalence was observed among calves aged ≤6 months (36.9%) compared with those aged >6–12 months (20.9%). This association was further supported by multivariable logistic regression analysis, which showed that calves aged ≤6 months had 5.73 times higher odds of infection than calves aged >6–12 months. The higher occurrence of coccidiosis among younger calves is consistent with previous studies reporting greater susceptibility of young animals to coccidial infection (Tamrat et al. 2020, Worku et al. 2019, Hussin 2016). The higher susceptibility of younger calves observed in the present study may be associated with the immunobiology of *Eimeria* infection. Protective immunity against *Eimeria* is species‐specific and develops gradually following repeated exposure to infective oocysts (Faber et al. 2002). Young calves have limited prior exposure and therefore have not yet acquired sufficient protective immunity. In contrast, older calves may develop partial immunity following repeated exposure to the parasite (Faber et al. 2002). Furthermore, coccidiosis may be self‐limiting, with recovery occurring after the parasite completes its endogenous developmental stages (Daugschies and Najdrowski 2005). These factors may partly explain the lower prevalence observed in older calves. However, other studies have reported higher prevalence in older calves than in younger calves (Raschke 2007; Mekonnin and Furgasa 2018; Dawid et al. 2012), which may be explained by differences in cumulative exposure to *Eimeria* spp. as calves age. These contrasting findings suggest that the relationship between age and infection may vary according to local ecological conditions and husbandry practices.

The farming system was significantly associated with the prevalence of calf coccidiosis. Calves reared under intensive farming systems had the highest prevalence (41.4%), followed by those under semi‑intensive (17.0%) and extensive systems (14.3%). Multivariable logistic regression analysis showed that calves managed under intensive systems had 7.34 times higher odds of infection compared with those managed under extensive systems, after adjusting for other variables. Similar associations were also reported by studies in other parts of Ethiopia (Asfaw et al. 2016; Mohamed et al. 2017). The higher prevalence in intensively managed calves may be associated with management conditions that facilitate exposure to infective oocysts, including animal concentration, housing hygiene, and the accumulation of faecal material. Higher stocking densities in intensive systems increase the probability of fecal–oral transmission by concentrating infectious oocysts in a smaller area (Keomoungkhoun and Taweenan 2015). Confined housing often results in poor ventilation and the accumulation of moisture in bedding and flooring, creating environmental conditions that favour oocyst sporulation (Mitchell et al. 2012). Additionally, the stress associated with intensive management may impair cell‐mediated immunity, increasing calves’ susceptibility to clinical disease (Lucas 2011). These findings highlight the importance of hygiene and calf‐housing conditions in intensive production systems, where higher animal density and environmental contamination increase infection pressure compared with extensive systems.

Coccidiosis prevalence was higher among calves with poor body condition (65.3%) than those with medium (23.5%) or good body condition (7.0%). Calves with poor body condition had 14.46 times higher odds of infection than well‐conditioned calves. Similar associations have been reported previously (Tamrat et al. 2020; Ambaw et al. 2025; Asfaw et al. 2016; Alemnew et al. 2017). The relationship between body condition and coccidiosis may be bidirectional, as poor condition may increase susceptibility to *Eimeria* infection through impaired immunity (Peek et al. 2018), while coccidiosis may also reduce body condition through intestinal damage and poor nutrient utilization (Bangoura and Bardsley 2020). However, due to the cross‐sectional design, the direction of this association cannot be determined and should be interpreted cautiously. This finding differs from a previous report showing higher prevalence in well‐conditioned calves, possibly due to differences in management and production systems (Worku et al. 2019).

Herd size was significantly associated with calf coccidiosis. The highest prevalence was observed in large herds (>50 animals) (55.2%), followed by medium (29.6%) and small herds (23.5%). Multivariable logistic regression analysis showed that calves from large herds had 9.79 times higher odds of infection compared with those from small herds after adjusting for potential confounding factors. Similar associations between larger herd size and increased coccidial infection have been reported in Holeta (Regasa et al. 2018) and Kenya (Gakuru et al. 2022). The increased odds of infection among calves from herds containing more than 50 animals may reflect greater opportunities for faecal contamination or differences in management conditions associated with herd size. Larger herds may have higher stocking densities, increasing the likelihood of fecal–oral transmission (Koutny et al. 2012). Additionally, more animals may contribute to greater environmental accumulation of *Eimeria* oocysts (Vilatuña et al. 2026). Maintaining consistent hygiene and adequate space for calves may also be more challenging in larger herds, potentially increasing exposure to infective oocysts (Rehman et al. 2011). However, herd size does not necessarily indicate overcrowding or poor management, and these potential mechanisms were not directly established by the present study. Therefore, herd size should be interpreted as an associated factor that may reflect differences in exposure and management rather than a direct cause of infection.

Floor type was significantly associated with calf coccidiosis. Calves housed on soil floors had a higher prevalence of infection (37.3%) than those kept on concrete floors (24.0%) and had 6.81 times higher odds of infection. Similar associations have been reported in previous studies (Lopez‐Osorio et al. 2020; Ashagrie et al. 2022; Rehman et al. 2011). The higher odds of infection among calves housed on soil floors may be associated with differences in moisture retention, faecal contamination, and ease of cleaning compared with concrete floors. These conditions may influence the persistence of *Eimeria* oocysts in the farm environment (Parker and Jones 1990). Soil floors may retain moisture and faecal material, potentially creating conditions that favour oocyst survival, whereas well‐maintained concrete floors may facilitate cleaning and removal of faecal contamination. This interpretation is consistent with epidemiological evidence linking non‐cemented floors to higher *Eimeria* prevalence (Sufi et al. 2016). However, the present study did not directly assess oocyst survival or viability on different flooring materials. Therefore, the findings highlight the importance of floor hygiene, adequate drainage, and regular removal of faecal material without establishing a direct causal effect of flooring type on infection.

Calves that accessed river water had a higher prevalence of coccidiosis (37.1%) than those using tap water (30.0%) or underground water (14.7%). Multivariable logistic regression analysis showed a significant association between river water use and *Eimeria* infection. This association is consistent with previous studies (Ashagrie et al. 2022; Abebe and Tadesse 2015). This association may reflect greater exposure to faecal contamination, as surface water can receive runoff from livestock areas, grazing lands, and surrounding settlements (Daugschies 2000). The environmental persistence of *Eimeria* oocysts may further contribute to potential exposure through contaminated water sources (Cruvinel et al. 2024). In contrast, properly treated tap water and protected underground water sources may reduce exposure to faecal contaminants (Lassen et al. 2014). However, water samples were not examined in the present study; therefore, contamination of river water with *Eimeria* oocysts and direct waterborne transmission could not be confirmed. These findings highlight the importance of access to safe drinking water as a potential component of calf coccidiosis prevention, while further studies incorporating water‐quality assessment are needed to clarify the role of water sources in transmission.

The results showed that non‐weaned calves had significantly lower odds of *Eimeria* infection than weaned calves (AOR = 0.19), indicating that non‐weaned calves had approximately 81.0% lower odds of infection compared with weaned calves. This finding is consistent with previous studies reporting that weaning stress can compromise immune function and increase susceptibility to *Eimeria* infection (Bruhn et al. 2011; Lucas et al. 2007; Parker et al. 1984). Weaning is a stressful management period involving dietary changes, separation from the dam, and exposure to new environments, which may reduce immune competence and increase vulnerability to infectious agents. Additionally, calves are often grouped during the weaning period, increasing the risk of fecal–oral transmission through greater animal contact and contamination of shared environments (Roth et al. 2009).

### 4.3 Farmers’ Knowledge and Farm Management Practices Related to Calf Coccidiosis

In addition to the associated factors analyzed, farmers’ knowledge and farm management practices related to calf coccidiosis were assessed to provide further insight into disease transmission and control. Knowledge of coccidiosis among farm owners was low, with only 15.5% of the respondents reporting prior knowledge of the disease. This finding agrees with previous reports from Ethiopia, where low levels of farmer knowledge and disease prevention practices have been documented across different livestock production systems (Yizengaw et al. 2026; Chere 2019; Ambaw et al. 2026). This limited knowledge has important implications for disease control. Limited awareness may hinder the recognition of potential disease signs and the adoption of appropriate preventive measures, although these effects were not directly assessed in the present study. Effective prevention of coccidiosis requires appropriate hygiene, proper waste disposal, and management practices that minimize calves’ exposure to faecal contamination (Mitiku et al. 2025). Therefore, improving farmers’ awareness of disease transmission, recognition, and prevention may contribute to better calf health management.

Proper waste disposal was practiced on less than half of the farms (46.5%), one‐quarter of farms did not provide colostrum feeding (25.4%), calf contact with adult cattle was common, and footbath use was largely absent (90.1%). Similarly, inadequate implementation of control measures was observed on more than half of the farms (54.9%), indicating a gap in the adoption of preventive strategies against coccidiosis. This finding is consistent with reports from other parts of Ethiopia, where poor biosecurity and limited adoption of disease prevention practices were common (Mitiku et al. 2025; Birhanu et al. 2025). These practices may increase environmental contamination with *Eimeria* oocysts and enhance exposure of susceptible calves to infection (Blowey and Weaver 2011). Furthermore, inadequate hygiene and biosecurity measures may facilitate the persistence and transmission of oocysts within farms (Campbell 2018). Similar deficiencies in calf management, hygiene, and biosecurity practices have been reported in other parts of Ethiopia (Belay and Mekibib 2022). Although the contribution of each management practice to infection was not independently established in this assessment, the observed deficiencies identify potentially modifiable areas for intervention. The identified gaps in farmers’ knowledge and farm management practices highlight the need for targeted farmer education and extension interventions to strengthen preventive practices and reduce the risk of calf coccidiosis in the study area.

### 4.4 Limitations of the Study

Several limitations should be considered when interpreting the findings. The cross‐sectional design precludes establishing temporal or causal relationships between *Eimeria* infection and the identified associated factors. *Eimeria* species were not identified, limiting species‐specific epidemiological interpretation and targeted control recommendations; species identification would have required additional procedures, including oocyst sporulation and detailed morphological examination, which were beyond the scope of the study given the available laboratory facilities. Although palpation was used to improve body condition scoring, the scoring criteria were not specifically validated for the local and crossbred calves included in the study. Variation in age, breed, and body composition may therefore have resulted in some misclassification, and the association between body condition and *Eimeria* infection should be interpreted with caution. Shared environmental exposures, such as communal grazing areas, watering points, and other commonly used resources, were not assessed and may have contributed to similarities in infection status among calves from different households, particularly under extensive production systems. In addition, some facility‐related variables, particularly housing conditions included in the analysis, were not directly comparable across production systems because extensive farms generally lacked confined housing facilities.

Despite these limitations, the study provides baseline information on *Eimeria* infection, associated factors, farmer awareness, and management practices, while informing future research and context‐specific control strategies.

## 5 Conclusion and Recommendations

This study found that *Eimeria* infection was common among calves in and around Gondar City, with a calf‐level prevalence of 29.7% and a farm‐level prevalence of 59.2%. Infection was significantly associated with age, body condition, weaning status, management system, herd size, floor type, and water source. These findings suggest that calf characteristics and farm management conditions may contribute to exposure to *Eimeria* infection. In addition, the study identified limited farmer awareness of coccidiosis and deficiencies in waste disposal, colostrum provision, and biosecurity practices on the surveyed farms. Collectively, these findings highlight the need to strengthen preventive management practices and farmer awareness to reduce calves’ exposure to *Eimeria* infection.

Based on the findings of this study, the following measures are recommended. Age‐targeted and management‐based coccidia control strategies are warranted, with particular emphasis on younger calves and those managed under intensive management systems. The association between river water use and infection highlights the need to promote safe water sources for calves, while further research is needed to confirm whether water quality directly contributes to *Eimeria* exposure. Floor hygiene should be improved through regular removal of faecal material, adequate drainage, and maintenance of clean and dry calf‐housing areas. Weaning management and space availability should also be reviewed, particularly in intensive production systems and larger herds, although the effectiveness of specific interventions targeting these factors requires further evaluation. Farmer education on coccidiosis recognition, transmission, prevention, colostrum provision, and effective hygiene practices should be strengthened in the study area. Further studies should identify *Eimeria* species, investigate seasonal and environmental exposures, assess water quality, and use longitudinal designs to clarify temporal relationships between infection and the associated factors identified in this study.

## Author Contributions

**Nibretu Tsegaye Guto**: conceptualization, sample collection and investigation, methodology, software, formal analysis, writing – original draft, writing – review and editing. **Yohannes Balework Zeleke**: data curation, methodology, software, writing – review and editing. **Gebremikael Asnakew Wubie**: conceptualization, data curation, writing – review and editing, project administration. **Bezawit Kassaw Hailu**: writing – original draft, resources, validation, visualization. **Ambaye Kenubih**: Conceptualization, investigation, methodology, software, supervision, writing – review and editing.

## Funding

The authors have nothing to report.

## Ethics Statement

This study was approved by the Animal Welfare and Ethical Review Committee of the College of Veterinary Medicine and Animal Sciences, University of Gondar (Ref. No. CVMASc/UoG/RERC/24/11/2024) on 24 November 2024. All appropriate precautions were taken to minimize pain experienced by animals in this study. Prior to data collection, informed consent was obtained from all farm owners after clearly explaining the purpose and procedures of the study. All methods were performed in accordance with relevant guidelines and regulations.

## Conflicts of Interest

The authors declare no conflicts of interest.

## Supporting information




**Supplementary material 1**: Description of condition scores
**Supplementary material 2**: Data collection sheet used during sample collection for individual calves.
**Supplementary material 3**: Laboratory procedures followed during fecal examination
**Supplementary material 4**: Coccidia oocysts examined under a microscope by fecal floatation
**Supplementary material 5**: Questionnaire prepared to assess farm management practices and demographic characterstics of farm owners.

## Data Availability

The data that support the findings of this study are available from the corresponding author upon reasonable request.
