# Supplementary material for: Prevalence, Associated Risk Factors, Farmers' Knowledge, and Farm Management Practices of Calf Coccidiosis in and Around Gondar City, Northwest Ethiopia
Source: Vet Med Sci. 2026 Nov 5;12(6):e71326. doi: 10.1002/vms3.71326 (PMC13739129; doi:10.1002/vms3.71326)
Supplement: Supplementary file 1 [file VMS3-12-e71326-s001.docx]

**Supplementary material 1:** Description of condition scores

| Score | Condition | Features |
| --- | --- | --- |
| 1 | Poor | Marked emaciation |
| 2 |  | Transverse processes project prominently, neural spines appear sharply |
| 3 |  | Individual dorsal spines are pointed to the touch, hips, pins, tail-headed, and ribs are prominent, transverse processes visible usually individually |
| 4 | Medium | Ribs, hips and spines clearly visible, muscle mass between hooks and pins slightly concave, slightly more flesh above the transperse process |
| 5 |  | Ribs usually visible, little fat cover, dorsal spines barely visible |
| 6 |  | Animal smooth and well covered; dorsal spines cannot be seen, but are easily felt. |
| 7 | Good | Animal smooth and well covered, but fat deposits are not marked. Dorsal spines can be felt with firm pressure, but fill rounded rather than sharp. |
| 8 |  | Fat cover in critical areas can be seen easily seen and felt; transverse processes cannot be be seen or felt |
| 9 |  | Heavy deposits of fat clearly visible on tail-headed, brisket and cod; dorsal spines, ribs, hooks and pins fully covered and cannot be felt even with firm firm preesure |

**Supplementary material 2**: Data collection sheet used during sample collection for individual calves

| No | Date | Calf ID | F. ID | F.SY | Sx | Age  (Mths) | BC | Breed | Fecal.cos | Reciently weaned? | Result |
| --- | --- | --- | --- | --- | --- | --- | --- | --- | --- | --- | --- |
| 1 | Jan/05/025 | C001 | F1 | Intens | M | 4 | good | cross | diarrheic | yes | +ve |
| 2 |  | C002 | F2 | Extensi | F | 3 | Poor | local | normal | No | -ve |
|  |  |  |  |  |  |  |  |  |  |  |  |
|  |  |  |  |  |  |  |  |  |  |  |  |
| 255 |  |  |  |  |  |  |  |  |  |  |  |
| 256 |  |  |  |  |  |  |  |  |  |  |  |
| Abbrevations: CO= Continue, F.ID= Farm identification, F.SY= Farming system, F.size= Farm size, BC= Body Condition, Sx= sex, M= male, F=Female, Mths= months, +ve= positive, -ve=negative | | | | | | | | | | | |

**Supplementary material 3**: Laboratory procedures followed during fecal examination

Faecal samples were processed using the floatation method according to the procedure described in The procedure in brief is:

(i) About 3 gram**s** of fresh fecal sample was collected and placed in a clean container.
(ii) Approximately 15–20 mL of saturated sodium chloride solution was added to the sample.
(iii) The feces were thoroughly mixed with the solution using an applicator stick to form a uniform suspension.
(iv) The suspension was filtered through a metallic sieve in to centrifuge test tube into a clean test tube and Floatation fluid was added into the test tube until slight convex meniscus formed at the top.
(v) Then cover slip was placed on the top of the tube, making sure no air bubbles were present and allowed to stand for 10 minutes.
(vi) The tube was allowed to stand undisturbed for 15–20 minutes.
(vii) The cover slip was then carefully lifted straight up and placed on a microscope slide.
(viii) The slide was examined under a microscope at 10× and 40× magnifications for identification of Eimeria oocysts.

**Supplementary material 4:** Coccidia oocysts examined under a microscope by fecal floatation

|  |  |
| --- | --- |
|  |  |

**Supplementary material 5**: Questionnaire prepared to assess farm management practices and demographic characterstics of farm owners

**A. General Information**

1. Farm Name: ______________________________________

2. Location (kebele): ___________________________

3. Date: ___________________________________________

4. Contact Person: __________________________________

5. Contact Information (phone No): _______________________________.

**B. Socio-demographic characteristics of farm owners**

6. Personal ID: ___________________________________________________.

7. Kebele/Peasant Association (PA): __________________________________.

8. Sex: 1. Male	2. Female

9. Age category in years: 1. 18-25	2. 26-30	3. 31-35	4. >35

10. Educational status: 1. Illiterate	2. Primary	3. Secondary	4. Diploma and above

11. Are you a veterinarian? 1. Yes	2. Not

12. Years of experience in dairy/ beef farming? ______________.

13. Did you get any training in dairy/ beef farming? 1. Yes	2. No

C. **Farm Characteristics**
17. Farm purpose: 1. Dairy	2. Beef	3. Both
18. What is your farming type? 1. Intensive	2. Semi-intensive	3. Extensive
19. What is your scale of production (in number of animals)?
1. 1-5	2. 6-10	3. 11-20	4. 20-50	5. >50
20. What type of housing do you provide for your calves?

1. Individual pens	2. Group housing	3. Pasture-based system	4. Other: ___________.

21. How many calves do you have? _______________________________.

22. What is the housing condition of your farm? 1. Indoor 2. Outdoor

23. House type: 1. Separated	2. Unseparated

23. Barn hygiene: 1. Poor	2. Good	3. Very good

23. What is the Floor type of your house? 1. Concert	2. Soil

24. What is the Feeding type of your calve?

1. Trough feeding	2. Concentrate feeding	3. Free grazing	4. Suckling

25. What Type of Bedding Options are used in your farms?

1. Sand	2. Sawdust	3. Straw	4. Paper	5. Others: ______________.

26. What is the Water source of the calves?

1. River water	2. Tap water	3. Underground water

27. If the answer to the above question is river water, is it treated or not? 1. Yes	2. No

28. If yes, what kind of treatment is used? ___________________________________.

D. **Management Practices**
34. How often do you clean the housing? 1. Daily	2. Once a Week	3. Monthly	4. Rarely

35. What type of feed do you provide to your calves?

1. Milk replacer	2. Whole milk	3. Grain-based feed	4. Forage	5. Other

36. What type of supplement is provided?

1. Hay	2. Cereal	3. Factory byproducts	4. Others:_______.

37. How often do you conduct health checks on your calves?

1. Daily	2. Weekly	3. Monthly	4. Never

39. What is manure management on your farm? 1. On farm	2. Off-farm

40. What type of manure management system do you currently use?

1. Composting, 2. Anaerobic digestion, 3. Direct land application

41. How often is manure removed from calf housing areas?

1. Daily	2. Once a week	3. Monthly	4. Rarely

42. Are there specific areas designated for manure storage? 1. Yes	2. No

43. If so, where it is stored?

1. Waste storage ponds	2. In above-ground tanks	3. Below ground storage tanks.

44. Have any calves shown signs of diarrhea or other gastrointestinal issues in the past month?

1. Yes	2. No

45. If your answer is yes, what type of measure have you taken? _________________________.

46. Do you use a foot bath? 1. Yes	2. No

47. If yes, what is the frequency you changed? 1. After each use 2. After several use

48. Also if your answer is yes to question 13, is it enough in volume (is it deep enough to submerge the feet comfortably up to the ankles)? 1. Yes	2. No

59. Do you disinfect the house? 1. Yes	2. No

50. If yes, how often do you disinfect?

1. Routine (weekly or bi-weekly)	2. Post-Outbreak Protocols	3. Seasonal

51. Do you vaccinate your cows? 1. Yes	2. No

52. Do you use separate footwear/shoes for farming? 1. Yes	2. No

53. Do you have a separate feed store? 1. Yes	2. No

54. Who takes care of the calf? 1. Owner	2. Employer

55. Is it possible to contact other farms/ neighbor herds? 1. Yes	2. No

56. Are new calves isolated before introducing them to the herd? 1. Yes	2. No

E. **Environmental Factors**

57. What is the average temperature range in your area during the calf-rearing period?

1. Below 0°C	2. 0°C-10°C	3. 11°C-21°C	4. Above 21°C

58. Humidity Levels in Housing Area: 1. Low (<30%) 2. Moderate (30%-60%) 3. High(>60%)

59. Presence of Other Livestock on the Farm: 1. Yes	2. No

60. If yes, what types of livestock are there? _________________________________________.

61. Do you have a drainage system in place to manage waste and runoff? 1. Yes	2. No

62. Is the drainage inadequate (are there puddles or poorly managed waste areas)? 1. Yes	2. No

33. If yes, do you implement effective drainage systems to reduce moisture levels? 1. Yes	2. No

64. Is there any animal that is diagnosed or/and treated for coccidian? 1. Yes	2. No

65. If yes, in which season were you most diagnosed?

1. Summer	2. Winter	3. Spring	4.Autoneum

66. Is there a sudden weather change in the last month? 1. Yes	2. No

F. **Knowledge and Awareness**
67. Can you describe any instances of calf diarrhea you have encountered on your farm? What do you think might have caused it? ____________________________.
68. Do you know *E. bovis*, the protozoan parasite belonging to the genus Eimeria?
1. Yes, 2. No
69. Are you aware of coccidiosis as a disease affecting calves? 1. Yes	2. No

70. If yes, are you aware of its transmission routes? 1. Yes	2. No

71. If your answer is yes to #3, do you know the symptoms of coccidiosis? 1. Yes	2. No

72. If yes, have you gained any information regarding coccidiosis prevention in dairy/ beef farming? 1. Yes	2. No

73. Is there any calf on your farm with coccidia/diarrhea? 1. Yes	2. No

74. If yes, what is the Degree of infection? 1. Mild, 2. Moderate	3. Sever

75. Is the severity of the disease associated with age? 1. Yes	2. No

76. Do you have Colostrum awareness? 1. Yes	2. No

77. If yes, do you know the Time of the first colostrum feeding? 1. Yes	2. No

78. If yes, what is the time of your first colostrum feeding for your calf after birth?

1. <3 hour	2. 3-6 hour	3. 6-12hour	4. >12hour
